# The Health Innovation Impact Checklist: a tool to improve the development and reporting of impact models for global health innovations

**DOI:** 10.1080/16549716.2022.2056312

**Published:** 2022-04-22

**Authors:** Minahil Shahid, Amy Finnegan, Kelly Kilburn, Krishna Udayakumar, Joy Noel Baumgartner

**Affiliations:** a Duke Global Health Institute, Duke University, Durham, North Carolina, USA; b IntraHealth International, Chapel Hill, North Carolina, USA; c Centers for Disease Control and Prevention, Atlanta, Georgia, USA; d Global Health Innovation Center, Duke University, Durham, North Carolina, USA; e School of Social Work, University of North Carolina at Chapel Hill, Chapel Hill, North Carolina, USA

**Keywords:** Global health, health innovation, impact modeling, checklist, cost-effectiveness analysis

## Abstract

Donor financing is increasingly relying on performance-based measures that demonstrate impact. As new technologies and interventions enter the innovation space to address global health challenges, innovators often need to model their potential impact prior to obtaining solid effectiveness data. Diverse stakeholders rely on impact modeling data to make key funding and scaling decisions. With a lack of standardized methodology to model impact and various stakeholders using different modeling strategies, we propose that a universal innovation impact checklist be used to aid in transparent and aligned modeling efforts. This article describes a new Health Innovation Impact Checklist (HIIC) – a tool developed while evaluating the impact of health innovations funded under the Saving Lives at Birth (SL@B) program. SL@B, a global health Grand Challenge initiative, funded 116 unique maternal and newborn health innovations, four of which were selected for cost-effectiveness analyses (CEAs) within our evaluation. A key data source needed to complete a CEA was the lives saved estimate. HIIC was developed to help validate draft impact models from the SL@B donors and our own team’s additional modeling efforts, to ensure the inclusion of standardized elements and to pressure test assumptions for modeling impact. This article describes the core components of HIIC including its strengths and limitations. It also serves as an open call for further reviewing and tailoring of this checklist for applicability across global efforts to model the impact of health innovations.

## Background

Estimating the health and economic impacts of a policy, program or project is critical for informing and scaling innovative healthcare solutions [1]. Donor funding and investments from multilateral, bilateral, and global health initiatives are increasingly relying on performance-based measures [2–5]. In addition, donor demands are shifting from technical and academic outputs to impacts that measurably benefit society [6]. To demonstrate efficiency and garner donor and private sector interest, global health implementing agencies need to be able to measure and report the impact of their interventions on health outcomes.

In the field of global health innovation, impact is not realized unless an innovation is successfully developed, taken to scale and demonstrates effectiveness [7]. However, with rapidly changing health markets, rigorous evaluations for new interventions are often too costly and time-consuming to conduct relative to decision-making timelines [8–10]. It is estimated that less than 5% of drug and/or technology innovations reach scale, while the rate of achieving scale is 14 years on average [11]. With such high levels of uncertainty and wait times, innovators need predictive modeling to estimate the efficiency and future impact of their innovation, enabling donors and investors to make key funding and scaling decisions prior to the availability of widespread effectiveness data.

Within the field of maternal, newborn, and child health (MNCH), a few impact modelling tools already exist. The Lives Saved Tool (LiST), developed at the Johns Hopkins Bloomberg School of Public Health, has been used to estimate the impact of scaling up interventions in low- and middle-income countries [12]. The tool uses ‘coverage’, or the population in need that receives an intervention, as a key input to calculate cause-specific mortality. The default coverage data for the tool comes from large-scale, nationally representative surveys such as Demographic and Health Surveys and Multiple Indicator Cluster Surveys [12,13]. PATH, a nonprofit global health organization, under its Innovation Countdown 2030 initiative modeled 11 interventions using LiST to learn that 6.6 million mothers and children could be saved between 2016 and 2030 if these innovations could be scaled up [14]. PATH’s modeling strategy also pivoted around ‘coverage’ to determine how an innovation could help expand access to basic health services [14].

Another non-profit global organization, Population Services International (PSI), developed its own modelling strategy for estimating the health impact of its product distribution and service delivery efforts [15]. Unlike PATH, PSI sought to understand the impact of a single product or service delivered by the organization, and wanted cross-country and cross-program comparisons. As a result, it adopted a disability adjusted life year (DALY) measure to calculate the number of healthy years of life not lost to disability or death due to a PSI service [15].

RTI International, with support from The Bill and Melinda Gates Foundation, has developed the Maternal and Neonatal Directed Assessment of Technology (MANDATE) model. MANDATE is a web-based tool to assess the impact of medical technologies on maternal, fetal and neonatal mortality in low-resource settings [16]. The model allows users to adjust variables related to a technology’s availability, appropriate use and efficacy to estimate the potential number of maternal and newborn lives saved [17,18].

Grand Challenges Canada (GCC) and Results for Development’s (R4D) has another impact modeling approach. GCC developed simple spreadsheet models to estimate the number of lives saved and lives improved due to health innovations funded under the Saving Lives at Birth (SL@B) program [7]. These interventions are novel and require the consideration of contextual factors and feasibility of scale-up as they have not demonstrated effectiveness at scale [19]. GCC could not use LiST or MANDATE to model its innovations’ impact as these tools do not account for context-specific modalities, while PSI’s DALY approach was inconsistent with GCC’s lives saved estimate [7]. GCC models are based on the innovation’s theory of change, which helps reveal the chain of events that connect the direct effect of the innovation to health outcomes. These chain of events under various scenario analyses, including different assumptions about the effectiveness of an intervention, form the key parameters that are included in the model [7].

With an increase in the use of innovation impact modeling and the diverse range of modeling methodologies being adopted by various organizations (including PATH, PSI, GCC, and the MANDATE initiative), there is a growing need for standardization and quality assurance. Compared with clinical studies that report the effectiveness of an intervention, health intervention impact modeling takes into consideration broader, system-level factors such as the baseline health status of a population benefitting from an intervention, local service delivery capacity, as well as implementation-related issues affecting intervention coverage rates. This results in a diverse range of modeling approaches with varying impact metrics, which can be challenging to review and compare against one another. For example, PATH and GCC both modeled the projected impact of the same innovation (new inhaled formulation of oxytocin, a gold standard therapy for post-partum hemorrhage that currently requires refrigeration and administration by injection) across a similar timeframe but reached diverging estimates – PATH estimated 146,000 [20] maternal lives saved between 2022 and 2030, while GCC estimated 27,000 [7] lives saved between 2020 and 2030, globally. The lack of standardization in modeling makes it difficult to tease out specific assumptions used by both organizations, which generated differences in their model outcomes.

Promoting transparency and comparability in impact modeling can potentially stem from the use of reporting guidelines and checklists. Evidence suggests that an endorsement of guidelines by journals can facilitate improved reporting [21]. Organizations using intervention impact modeling usually seek to project the impact of their interventions without necessarily having engaged in complex and large studies and can face challenges in data quality [22]. Guidelines and checklists can assist organizations in ensuring a minimum standard of reporting.

Duke University was engaged in 2018 by the USA Agency for International Development (USAID) and GCC – two SL@B funding partners – to design and conduct an evaluation of the SL@B program to determine if it was achieving its intended impact. The SL@B program has issued 147 awards representing 116 unique innovations and 92 organizations addressing critical issues in maternal and newborn health (MNH) in low-resource settings [23]. One component of the evaluation required estimating the potential impact of SL@B-funded innovations on maternal and neonatal mortality, which included reviewing impact models developed by GCC and R4D for four interventions. During this review process, Duke University’s Evidence Lab team referred to academic literature to find pre-existing and widely recognized tools and guidelines that could assist them in their validation efforts. Being unable to find one relevant and standardized tool, the team developed its own checklist for health impact models, henceforth referred to as the Health Innovation Impact Checklist (HIIC). Although initially developed to complement our review of GCC’s models, HIIC was further developed to provide a generalized reference or guide for various types of innovation impact models for our broader work with global innovators and attempts to consolidate and standardize multiple modeling approaches. The following section will introduce and explain the HIIC using some examples from innovations funded by SL@B.

## The Health Innovation Impact Checklist (HIIC)

The HIIC is a tool to help review the standardized elements and pressure test assumptions of impact models. The checklist (see Table 1) is a qualitative tool designed to review quantitative models and can be used by both reviewers and developers of health intervention impact models to help strengthen their analyses.

**Table 1.** Health Innovation Impact Checklist (HIIC)

		Parameters Included		Sensitivity Assessed	
Section/Topic	Checklist item	Y/N/NA*	Describe	Y/N/NA	Describe
Model Description					
Theory of Change	Describe the innovation’s Theory of Change (TOC) or logic model.	*e.g. ‘Y’*	*Innovation has a clear logic model included in grant proposal*	*e.g. ‘N/A’*	
Study perspective	Describe the perspective of the study and relate this to the impacts being evaluated. Options include: innovator perspective, societal perspective, health/epidemiological perspective. (implications for cost-effective analyses)	*e.g. ‘Y’*	*Modeling focused on health sector perspective*	*e.g. ‘N/A’*	
Time horizon	State the time horizon(s) over which impact is being evaluated and describe why appropriate.	*e.g. ‘Y’*	*e.g. ‘we are modeling impact between 2014 – the year we launched the innovation – and 2030 – the SDG timeline’*	*e.g. ‘N’*	*e.g. ‘we are only interested in gauging the short-term impact of the innovation’*
Target population	Describe the target population.	*e.g. ‘Y’*	*e.g. ‘pregnant women with health problem X’*	*e.g. ‘N/A’*	
Assumptions					
*Demographic factors*					
Disease burden	Does the model include assumptions for how the at-risk population will change over time without the intervention? Describe whether the assumption is based on a level or trend change or both. For example, fertility trends, neonatal and maternal mortality trends etc. Describe the source of information used.	*e.g. ‘Y’*	*e.g. ‘this is the data on disease burden for pregnant women with health problem X based on limited evidence’*	*e.g. ‘Y’*	*e.g. ‘the data for this health problem are variable and thus we model both an upper estimate and lower estimate of the disease prevalence’*
*Efficacy and Fidelity*					
Efficacy	Does the model account for the efficacy of the innovation with typical use and perfect use compared to some counterfactual? Describe the source of information used. State the rigor of the evidence base for efficacy measurements. Options include: pilot, RCT, observational study (case-control), anecdotal evidence. Describe the sample size, p-value, effect size, level/tier of health system included in evidence, etc. Describe the source of information used.				
Real-world fidelity to treatment protocol (intermediary)	Does the model include parameters for provision of and quality of service delivery by intermediaries (e.g. health workers, manufacturing, facility infrastructure, etc.)? Describe the source of information used.				
Real-world fidelity to treatment protocol (beneficiary)	Does the model include parameters for fidelity to treatment protocol by the beneficiary (e.g. improper/sub-optimal use or engagement with innovation) ? Describe the source of information used.				
*Health system factors*					
Equitable access	Does the model include a parameter for equitable access to services? For example, does access to innovation vary across rural vs. urban populations, socio-economic status, other vulnerabilities or inequities? Describe the source of information used.				
Supply Chain	Does the model account for issues in supply chain and delivery (e.g. stock outs)? Describe how the model takes this into consideration and if not, why not. Describe the source of information used.				
Referrals	Does the model account for referral of severe cases? For example, what proportion of cases will be referred and once referred what proportion will actually reach care. Describe how the model takes this into consideration and if not, why not. Describe the source of information used.				
Attrition of intermediaries	Does the model include attrition and replacement of community health workers, nurses, etc.? Describe how the model takes this into consideration and if not, why not. Describe the source of information used.				
Scenarios					
Innovator scale-up	Does the model include innovator reported scale up as a scenario? Describe the scenario, including any assumptions about implementation challenges.				
Universal health coverage	Does the model include universal health coverage as a scenario? Describe the scenario, including any assumptions about implementation challenges.				

* Y: yes, N: no, N/A: not applicable; *Developed by the Duke Global Health Institute Evidence Lab at Duke University*

HIIC is comprised of three sections: 1) Model Description, 2) Assumptions and 3) Scenarios, and each section consists of multiple categories, henceforth referred to as *parameters*. Each parameter or a measurable element or factor that forms part of the checklist, highlights a particular aspect of an impact model, which the HIIC user gauges as relevant or applicable to their model or not. A parameter, in the context of this checklist, can consist of a single indicator (e.g. the ‘time horizon’ parameter can be a single year (e.g. 2030)) or a range of estimates (e.g. the ‘efficacy’ parameter can comprise multiple studies that demonstrate the effectiveness of the innovation under different settings). HIIC does not dictate how to create an impact model, instead it enables the user to review their own model against each parameter. HIIC also requires reviewing sensitivities in model estimates. Sensitivity analysis helps determine the robustness of a model by examining to what degree the model results are affected by changes in inputs or assumptions. By requiring the user to identify and explain the various model parameters and confidence intervals/sensitivities of model outcomes, HIIC promotes transparency in results and comparability across different impact models.

The HIIC Model Description section highlights the basic components of intervention impact models, including the *theory of change*, or the chain of events that connect the direct effects of an innovation to health outcomes. Mapping out this chain of events helps reveal the key measures that determine an innovation’s potential impact. Identifying these measures helps the reviewer or modeler gauge the inherent assumptions their model make. For example, the direct effect of a newborn temperature measurement device, known as the BEMPU TempWatch [24], would be an increase in the number of identified hypothermia cases that would not have been identified and treated in the absence of the TempWatch. The outcome, for example the number of newborn lives saved, will depend on many assumptions including but not limited to the number of newborns receiving access to the device, newborns using the device, and newborns receiving treatment after identification of hypothermia, all of which form the key measures to gauge the projected impact of BEMPU’s TempWatch [24].

The HIIC description section also highlights the following: *time horizon* of the model or the number of years across which impact is being measured (SL@B used 2030 as the end year to project impact, corresponding with the Sustainable Development Goals’ (SDGs) timeline); *target population* that will use an intervention or for whom the intervention will have an effect (e.g. the population of interest in the case of the BEMPU TempWatch is low birth weight newborns who are more likely to develop hypothermia than normal weight babies, particularly in lower resource settings) [24,25]; and the *study perspective*, which determines from whose standpoint the modeling exercise is being conducted. The perspective of a model may be one or more of the following: societal, healthcare sector, health practitioners, patients, innovators, and funding agencies supporting the development of an innovation and others. The impact of an intervention cannot be realized in the same manner across different perspectives due to divergent interests, making it crucial to identify which perspective to model from the outset. For example, a narrow perspective, such as that of health providers’, will not account for the use of resources outside the health sector or the greater welfare to society, which will be captured under the societal perspective. Considering that economic resource availability and output of any society are limited, improving healthcare via novel innovations will require devoting more resources to health, which may necessitate forgoing benefits or opportunities in other sectors [26].

The Assumptions section is subdivided along demographic factors, efficacy and fidelity of the innovation, and health system factors. *Demographic factors* refer to the context in which an innovation is being implemented and includes the *disease burden* parameter. The goal of this parameter is to question whether the model considers the baseline demographic factors, such as fertility trends, neonatal mortality or maternal mortality in the beneficiary population and models how these factors might change in the absence of innovation. Including trends in demographics and disease burden helps ensure that the model does not overestimate the potential future impact of the innovation.

The *efficacy and fidelity to treatment* sub-section includes the evidence base of the model and the protocols of real-world implementation of an intervention. The *efficacy* parameter gauges the effectiveness of an intervention during typical and perfect use and compares these against a counterfactual (hypothetical alternative to actual conditions minus the intervention) [27]. The effectiveness of an innovation oftentimes is the single most important factor that determines how impactful the intervention will be, making this parameter’s quality of evidence base an imperative task. Randomized controlled trials and propensity score matching studies in peer-reviewed journals are typically the ideal form of evidence, but other sources such as intervention pilots or long-term studies can also be quoted with additional discounting to account for limitations. Likewise, reliance on non-peer reviewed reports for parameter data is often necessary. By requiring the user of the checklist to report their sources of evidence, HIIC promotes transparency and allows internal and external reviewers of impact models to review and compare model data, assumptions and sources across different models.

The *fidelity to treatment* parameter reviews the process of intervention development and administration from a service delivery perspective. This parameter evaluates evidence on the ease of development, implementation and use of intervention by intermediaries, including manufacturers, health professionals, caregivers, and beneficiaries. For example, modeling the impact of Pratt Pouch, an innovation that delivers Nevirapine (NVP) (an antiretroviral prophylaxis) in a small sachet to HIV-exposed infants, requires taking into account the ‘correct’ use of the pouch [28]. Mothers can mistakenly fail to empty the complete contents of the pouch into an infant’s mouth or not complete the entire six-week NVP regimen, resulting in incorrect use, which can reduce the effectiveness of and thus negatively affect the innovation’s impact [28].

The Health System Factors section identifies key components of the overarching health system in which an intervention is being implemented, which can affect an innovation’s utility and potential impact. The health system should be inclusive of all potential implementation challenges given current human and resource constraints. If an intervention is in the form of a product or its functioning depends on the availability of certain equipment or tools at a health facility, then the *supply chain* parameter gauges if the model takes into account the availability of relevant equipment, tools, or products to enable intervention use. If the intervention is a service and requires trained healthcare staff to administer it, then the *attrition of health intermediaries* parameter checks if the model incorporates the regular turnover of health workers, which can decrease the knowledge of and use of an intervention. If the intervention does not cater to severe cases that need referral or only caters to highly severe referred cases, then the *referral* parameter requires that the model discounts the patients that the intervention does not serve. Access to the health intervention may also vary across different segments of the population such as urban versus rural residents or those in different wealth quintiles (economic status), etc. The *equitable access* parameter reports if these differences in access have been incorporated in the model.

The Scenarios section identifies the overarching expansion strategy for the innovation and enables the reviewer to report on intervention scale-up. If the innovation is modeled to achieve universal coverage in a certain country (versus, for example, scale in government or private sector health facilities only) then all other parameters must also follow suit. For example, the time horizon should reflect the time needed to scale-up across the country, while the disease burden parameter must identify fertility or mortality trends across the entire country’s population.

## Discussion

One of the key strengths of the HIIC is that it brings consistency and transparency across different kinds of intervention impact models. In the field of global health innovation where a diverse range of modeling methodologies are being adopted by different organizations, HIIC enables comparison between key elements of the models. Ensuring comparability and standardized approaches for impact modeling should help donors and governments as they seek to invest in and report out on effective and efficient innovations to achieve targeted health outcomes. For innovators and delivery organizations, comparability in modeling can facilitate a deeper understanding of their innovation’s performance by helping them identify parameters against which their innovations may be suboptimal.

In the health innovation field, where the effectiveness base for early-stage innovations can be missing or lacking strong evidence, a tool such as HIIC can help strengthen the analysis by enabling modelers to be more systematic and transparent while developing their estimates. HIIC is by no means a replacement for strategies already being used to model impact where effectiveness data already exist, but rather it helps to ensure more accurate and comparable estimates prior to the availability of that data. Using HIIC enables the modeler to state the assumptions that their model makes and justify the sensitivities used, making it easier for the modelers and reviewers studying the models to understand the rationale behind the estimates. HICC should help in communication between different organizations as they model impact so that any divergent estimates can be more easily explained and clarified.

Modeling the impact of health innovations is by nature a complex task. Each impact model presents its own unique measurement challenges, and requires quantification of diverse input, output and outcome measures. This complexity in design, however, is not replicated in HIIC, which cannot fully encompass all aspects of a model or dictate a particular framework that a model must use. Instead, HIIC only poses questions and highlights key parameters, which the user of the checklist determines whether they apply to their model or not.

Some parameters included in HIIC such as ‘efficacy’ and ‘equitable access’ can prove to be difficult to measure and require in-depth knowledge of an innovation and its implementation. Including these parameters, however, is critical for assessing impact. Despite strong estimates not being easily accessible, reporting on these parameters in HIIC will ensure that the strength of the evidence is captured, which can ultimately inform a reviewer about the robustness (or limitations) of a particular impact model.

## Conclusion

To make HIIC more comprehensive and potentially increase its user base, the next steps would entail engaging impact modelers who are using modeling techniques to review and critique HIIC. Field-testing HIIC on a range of impact models, keeping track of iterations resulting from this exercise and disseminating key learnings and revised versions of the checklist can add significant contribution to this trending field of innovation impact modeling. Differential weighting of parameters and their evidence base, scoring, and creating a more comprehensive list of essential and elective model components are examples of what future iterations of HIIC might look like. This article serves as an open call to further review and tailoring of this tool for applicability across global efforts to model the impact of health innovations.

## Paper context

There are varying global health innovation impact modeling approaches being used to demonstrate impact. A lack of standardization across these methodologies can create issues of transparency and comparability. The newly developed Health Innovation Impact Checklist (HIIC) is a qualitative tool for reviewing quantitative models, designed for modelers and reviewers to pressure test assumptions and review the standardized elements of health innovation impact models. We invite stakeholders who estimate innovation impact to further refine and test this checklist.

## References

[1] Herrick T, Harner-Jay C, Shaffer C, et al. Modeling the potential impact of emerging innovations on achievement of Sustainable Development Goals related to maternal, newborn, and child health. Cost Eff Resour Alloc. 2017;15:12. 28706466 10.1186/s12962-017-0074-7 PMC5506623

[2] Bertone MP, Falisse JB, Russo G, et al. Context matters (but how and why?) A hypothesis-led literature review of performance based financing in fragile and conflict-affected health systems. PLoS ONE. 2018;13:e0195301–8. 29614115 10.1371/journal.pone.0195301 PMC5882151

[3] World Bank [Internet]. Program-for-results financing (PforR); [cited 2021 Sept 27]. Available from: https://www.worldbank.org/en/programs/program-for-results-financing

[4] USAID & Palladium [Internet]. Pay for results in development: a primer for practitioners; [cited 2021 Sept 27]. Available from: https://www.usaid.gov/sites/default/files/documents/1865/Pay_for_Performance_Primer_Final.pdf

[5] The Global Fund [Internet]. The global fund strategy 2017-2022; [cited 2021 Dec 2]. Available from: https://www.theglobalfund.org/media/2531/core_globalfundstrategy2017-2022_strategy_en.pdf

[6] Graham KER, Langlois-Klassen D, Adam SAM, et al. Assessing health research and innovation impact: evolution of a framework and tools in Alberta, Canada. Frontiers in Research Metrics and Analytics. *Front Res Metr Anal*. 2018;3. DOI:10.3389/frma.2018.00025.

[7] The International Development Innovation Alliance (IDIA) [Internet]. Insights on measuring the impact of innovation; 2017 [cited 2021 Sept 27]. Available from: https://www.unhcr.org/innovation/wp-content/uploads/2018/06/Measuring20Impact.pdf

[8] Bennett S, Lagomarsino G, Knezovich J, et al. Accelerating learning for pro-poor health markets. Glob Health. 2014;10:54. 10.1186/1744-8603-10-54 PMC4105125 24961671

[9] Bhattacharyya O, Mossman K, Ginther J, et al. Assessing health program performance in low- and middle-income countries: building a feasible, credible, and comprehensive framework. Glob Health. 2015;11:51. 10.1186/s12992-015-0137-5 PMC4687324 26690660

[10] Langsam K, Clark C. Decoding the ABCs of effective enterprise acceleration: 10 lessons from the social entrepreneurship accelerator at duke (SEAD). Duke University; 2020 [cited 2021 Sept 27]. [Internet]. Available from: https://centers.fuqua.duke.edu/case/wp-content/uploads/sites/7/2020/10/Decoding-the-ABCs-of-Effective-Enterprise-Acceleration_Oct-12.pdf

[11] Gupta A, Thorpe C, Bhattacharyya O, et al. Promoting development and uptake of health innovations: the Nose to Tail Tool. F1000Res. 2016;5. DOI:10.12688/f1000research.8145.1 PMC4863676 27239275

[12] Winfrey W, McKinnon R, Stover J. Methods used in the lives saved tool (LiST). BMC Public Health. 2011;11:S32. 21501451 10.1186/1471-2458-11-S3-S32 PMC3231906

[13] Fox MJ, Martorell R, van den Broek N, et al. Assumptions and methods in the Lives Saved Tool (LiST). BMC Public Health. 2011;11:I1. 10.1186/1471-2458-11-S3-I1 PMC3231881 21501425

[14] PATH [Internet]. Harnessing the power of innovation to save mothers and children: how 11 innovations could save more than 6 million lives; 2016 [cited 2020 Sept 28]. Available from: https://www.path.org/resources/harnessing-the-power-of-innovation-to-save-mothers-and-children-how-11-innovations-could-save-more-than-6-million-lives/

[15] Yang H, Duvall S, Ratcliffe A, et al. Modeling health impact of global health programs implemented by Population Services International. BMC Public Health. 2013;13:S3. 10.1186/1471-2458-13-S2-S3 PMC3684543 23902668

[16] Healthy Newborn Network [Internet]. MANDATE: mobilizing innovations for maternal and neonatal health; 2011 [cited 2021 Nov 3]. Available from: https://www.healthynewbornnetwork.org/blog/mandate-mobilizing-innovations-for-maternal-and-neonatal-health/

[17] McClure EM, Rouse DJ, MacGuire ER, et al. The MANDATE model for evaluating interventions to reduce postpartum hemorrhage. Int J Gynaecol Obstet Off Organ Int Fed Gynaecol Obstet. 2013;121:5–9. 10.1016/j.ijgo.2012.10.030 PMC3628756 23313144

[18] RTI International [Internet]. Maternal and neonatal directed assessment of technology (MANDATE); [cited 2021 Nov 3]. Available from: http://www.mandate4mnh.org/

[19] Grand Challenges Canada [Internet]. Evidence based innovation annual report (2015-2016); [cited 2021 Dec 2]. Available from: https://www.grandchallenges.ca/wp-content/uploads/2016/03/Annual_Report_EN_Sept2016.pdf

[20] PATH [Internet]. The IC 2030 report: reimaging global health; 2015 [cited 2021 Sept 27]. Available from: https://www.pqmd.org/wp-content/uploads/2015/09/ic2030-Report-2015.pdf

[21] Turner L, Shamseer L, Altman DG, et al. Does use of the CONSORT Statement impact the completeness of reporting of randomised controlled trials published in medical journals? A Cochrane review. Syst Rev. 2012;1:60. 23194585 10.1186/2046-4053-1-60 PMC3564748

[22] David PH. Introduction to use of health impact metrics for programmatic decision making in global health. BMC Public Health. 2013;13:S1,1471–2458-13-S2-S1. 10.1186/1471-2458-13-S2-S1 PMC3684554 23902641

[23] Biru B, Rajan S, Taylor A, et al. Evaluating saving lives at birth (SL@B): final evaluation report of round 1 through round 8 (2011-2020) [Internet]. 2020 [cited 2021 Dec 2]. Available from: https://dukeghic.org/wp-content/uploads/sites/20/2020/07/E-SL@B-Final-Evaluation-Report.pdf

[24] Dixit S, Ogbuoji O, Fernholz F, et al. Evaluating saving lives at birth cost-effectiveness analysis: BEMPU-TEMPWATCH [Internet]. 2020 [cited 2020 Oct 13]. Available from: https://sites.globalhealth.duke.edu/evidencelab/wp-content/uploads/sites/23/2018/07/Bempu-CEA-Report.pdf

[25] Kumar V, Shearer JC, Kumar A, et al. Neonatal hypothermia in low resource settings: a review. J Perinatol. 2009;29:401–412. 19158799 10.1038/jp.2008.233

[26] Byford S, Raftery J. Economics notes: perspectives in economic evaluation. BMJ. 1998;316:1529–1530. 9582152 10.1136/bmj.316.7143.1529 PMC1113167

[27] Maldonado G. Estimating causal effects. Int J Epidemiol. 2002;31:422–438. 11980807

[28] Dixit S, Ogbuoji O, Fernholz F, et al. Evaluating saving lives at birth cost-effectiveness analysis: Elizabeth Glaser pediatric AIDS foundation (EGPAF) - Pratt Pouch [Internet]. 2020 [cited 2020 Oct 13]. Available from: https://sites.globalhealth.duke.edu/evidencelab/wp-content/uploads/sites/23/2018/07/EGPAF-CEA-Report.pdf

